# Predicting Which Mitophagy Proteins Are Dysregulated in Spinocerebellar Ataxia Type 3 (SCA3) Using the Auto-p2docking Pipeline

**DOI:** 10.3390/ijms26031325

**Published:** 2025-02-04

**Authors:** Jorge Vieira, Mariana Barros, Hugo López-Fernández, Daniel Glez-Peña, Alba Nogueira-Rodríguez, Cristina P. Vieira

**Affiliations:** 1 Instituto de Investigação e Inovação em Saúde (i3S), Universidade do Porto, Rua Alfredo Allen 208, 4200-135 Porto, Portugal; jbvieira@i3s.up.pt (J.V.); mariana-c-barros@hotmail.com (M.B.); alnogueira@uvigo.gal (A.N.-R.); 2 Instituto de Biologia Molecular e Celular (IBMC), Rua Alfredo Allen, 208, 4200-135 Porto, Portugal; 3 Faculdade de Ciências, Universidade do Porto, Rua do Campo Alegre s/n, 4169-007 Porto, Portugal; 4 Department of Computer Science, CINBIO, ESEI—Escuela Superior de Ingeniería Informática, Universidade de Vigo, 32004 Ourense, Spain; hlfernandez@uvigo.gal (H.L.-F.); dgpena@uvigo.gal (D.G.-P.); 5 SING Research Group, Galicia Sur Health Research Institute (IIS Galicia Sur), SERGAS-UVIGO, 36213 Vigo, Spain

**Keywords:** SCA3/MJD, mitophagy, in silico, pipeline

## Abstract

Dysfunctional mitochondria are present in many neurodegenerative diseases, such as spinocerebellar ataxia type 3 (SCA3), also known as Machado–Joseph disease (MJD). SCA3/MJD, the most frequent neurodegenerative ataxia worldwide, is caused by the abnormal expansion of the polyglutamine tract (polyQ) at ataxin-3. This protein is known to deubiquitinate key proteins such as Parkin, which is required for mitophagy. Ataxin-3 also interacts with Beclin1 (essential for initiating autophagosome formation adjacent to mitochondria), as well as with the mitochondrial cristae protein TBK1. To identify other proteins of the mitophagy pathway (according to the KEGG database) that can interact with ataxin-3, here we developed a pipeline for in silico analyses of protein–protein interactions (PPIs), called auto-p2docking. Containerized in Docker, auto-p2docking ensures reproducibility and reduces the number of errors through its simplified configuration. Its architecture consists of 22 modules, here used to develop 12 protocols but that can be specified according to user needs. In this work, we identify 45 mitophagy proteins as putative ataxin-3 interactors (53% are novel), using ataxin-3 interacting regions for validation. Furthermore, we predict that ataxin-3 interactors from both Parkin-independent and -dependent mechanisms are affected by the polyQ expansion.

## 1. Introduction

Spinocerebellar ataxia type 3 (SCA3), also known as Machado–Joseph disease (MJD) [1,2], is the most frequent autosomal dominantly inherited neurodegenerative ataxia worldwide [3]. It causes progressive cerebellar ataxia, which results in a lack of muscle control and coordination of the upper and lower extremities. It is caused by an abnormal polyglutamine (polyQ) expansion (over a threshold of 40 glutamines) at ataxin-3 [4], a deubiquitinating enzyme (DUB) that binds and cleaves polyubiquitin chains, thus modulating substrate degradation via proteasomal or autophagosomal machineries (for a review see [5]). polyQ is located at the C-terminal region (residues 292–305 in reference protein UniProt P54252), while the deubiquitinating function is encoded by the catalytic Josephin domain (JD) located at the N-terminal region (residues 1–180), but also by the three ubiquitin-interacting motifs (UIMs) located at the C-terminal tail (residues 224–243; 244–263; and 331–349 in reference protein UniProt P54252). The ataxin-3 polyQ region is involved in the stabilization of protein–protein interactions (PPIs) [6,7]. Abnormal polyQ expansion results in structural changes of the protein, implying different levels of accessibility at specific interacting residues and/or binding strength differences that compromise normal protein activity [7,8]. Given the large protein network of ataxin-3, numerous cellular functions can be, in principle, affected. An expanded polyQ region also promotes protein misfolding, resulting in protein aggregation and the formation of intracellular inclusions in specific brain regions of patients with SCA3/MJD, a pathological hallmark of the neurological disease [9,10]. Although it is not clear if these aggregates directly lead to toxicity [5,11], reducing the mutant ataxin-3 levels leads to fewer protein aggregates and to the preservation of neuronal cells [12].

Neuronal function is highly dependent on mitochondria, as neurons have a high energy demand [13]. In healthy cells, in order to maintain cellular homeostasis, damaged or dysfunctional mitochondria must be eliminated by a special autophagy process (mitophagy) that mediates the selective elimination of damaged, aged, or superfluous mitochondria. This process requires two major degradation systems, namely autophagy and the ubiquitin proteasome system (UPS) [14]. Mitochondrial dysfunction is observed in a number of diseases, such as neurodegenerative diseases [15], as well as in cardiovascular diseases, cancer, and metabolic diseases [16,17,18]. Although ataxin-3 is predominantly localized in the cytoplasm and nucleus, both its mutant expanded and wild-type (WT) forms have been associated with mitochondria [19,20], and, therefore, may directly influence mitochondrial function under normal and disease conditions. Moreover, ataxin-3 interactors are enriched in mitochondrial proteins [20], and a knock-in mouse model of SCA3/MJD (Ki91) shows dysfunctional energy metabolism [21]. Decreased cerebellar mitochondrial respiration and ATP production have also been described in another SCA3/MJD mouse model (MJD135), as well as in cells expressing mutant ataxin-3 (PC6-3) [22]. Furthermore, the isolated mitochondria of both mutant mice and PC6-3 cells show a significant decrease in their cytochrome c (that plays a critical role in cellular respiration) levels [22]. The above evidence suggests that, in SCA3/MJD, there is mitophagy dysregulation. A decrease in mitochondrial DNA has been observed in a mouse model (CMVMJD94; [23,24]) and one cell line (SK-N-SH MJD; [25]), as well as in the blood samples of patients with SCA3/MJD [25], but not in another mouse model (MJD135) or PC6-3 Q108 cells [22]. The identification of the key players that promote mitophagy in SCA3/MJD could offer new potential therapeutic opportunities.

**Figure 1:**
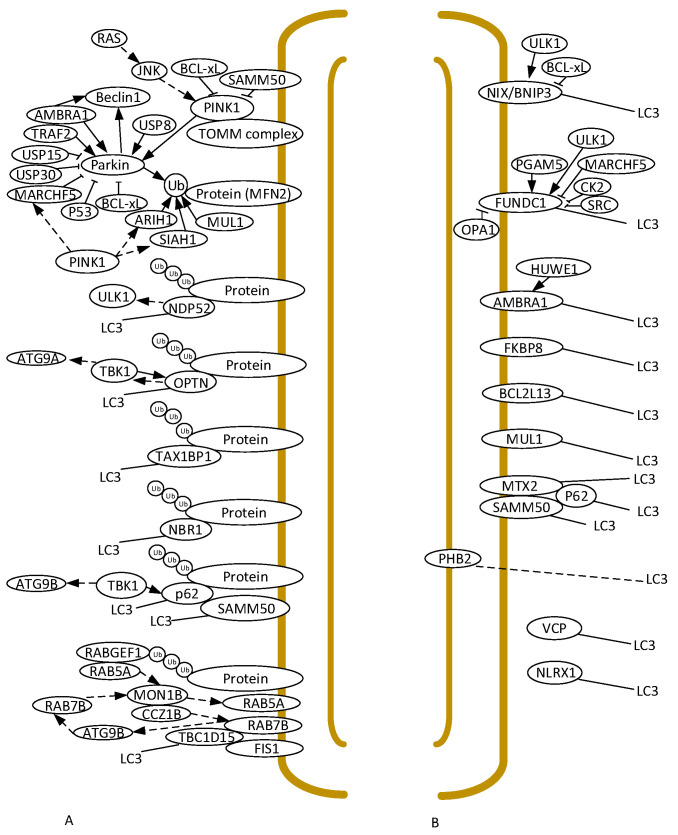
Ubiquitin-dependent (**A**) and -independent (**B**) mitophagy pathways in animals. Brown lines represent the mitochondrial membranes. Indirect interactions are marked with dashed lines.

Mitophagy can be dependent or not on E3 ubiquitin ligases, such as Parkin. In Parkin/PINK1-dependent mitophagy (see Figure 1A and Table S1 for full names), the Parkin activity is dependent on the PINK1 activity [14]. In healthy mitochondria, PINK1 is imported into the mitochondria and rapidly degraded by proteolysis. In damaged mitochondria, however, PINK1 is unable to be imported, and stays on the outer mitochondrial membrane (OMM), where it forms a high-molecular-weight complex with TOMM machinery, where it interacts with TOM7, which is a modulator of Parkin recruitment [26]. PINK1 stabilization in the OMM is regulated by JNK [27]. RAS has recently been found to activate mitophagy through the activation of JNK signaling [28]. The stabilization of PINK1 at the OMM leads to the subsequent phosphorylation and recruitment of Parkin [14]. Parkin activation is accompanied by a robust increase in its autoubiquitination that delays both its recruitment to damaged mitochondria and the successful completion of mitophagy [29]. USP8, a deubiquitinating enzyme, removes the ubiquitin chains from Parkin, allowing it to ubiquitinate its substrates (Figure 1A).

In mitochondria, there are four recognized Parkin substrates: MARF, MFN1, MFN2, and VDAC1 [14]. As a Parkin receptor, MFN2 can induce the migration of Parkin to damaged mitochondria, and PINK1/Parkin-mediated MFN2 ubiquitination may also induce mitophagy [30].

Beclin1 initiates the formation of autophagosomes. It has been suggested that Parkin interacts with Beclin1 (Figure 1A) to ensure their concurrent delivery to dysfunctional mitochondria upon the activation of mitophagy [31]. Another mitophagy receptor, AMBRA1, that interacts with Beclin1, inducing its activity, can also induce Parkin-dependent mitophagy, since, after mitochondrial depolarization, it can also interact with Parkin, enhancing mitochondrial clearance [32]. Nevertheless, the LIR region in AMBRA1 can also bind the autophagosome adapter LC3 during mitophagy induction (Figure 1B), and, thus, can also induce Parkin-independent mitophagy [33].

USP15 and USP30, which are deubiquitinating enzymes, counteract the Parkin-mediated mitochondrial ubiquitination of OMM proteins by removing ubiquitin chains attached by Parkin (Figure 1A), thus blocking mitophagy [34,35].

Stabilized PINK1 also recruits ubiquitin ligases such as MARCHF5 [36], which binds to Parkin and adds polyubiquitin chains to it, thus promoting Parkin degradation (Figure 1A). Moreover, SAMM50 interacts with PINK1 to facilitate its processing and degradation [37,38]. MARCHF5 can also bind to FUNDC1 (Figure 1B), promoting its degradation and thus decreasing mitophagy in a Parkin-independent way [36]. TRAF2, also an E3 ubiquitin ligase, interacts with Parkin (Figure 1A) to facilitate the ubiquitination of proteins on damaged mitochondria and assist in their removal through the process of autophagy [39].

P53 binds to Parkin (Figure 1A), thus inhibiting its recruitment to mitochondria [40,41]. BCL-xL also inhibits PINK1/Parkin-dependent mitophagy by physically binding to Parkin and forming oligomers in cytoplasm, thus preventing the recruitment of Parkin to the OMM. BCL-xL can also directly interact with PINK1 located at the OMM, thus interfering with the recruitment of Parkin by PINK1 to the OMM [42].

Ubiquitin ligases other than Parkin have been identified, as well, as playing a key role in mitophagy. Among them, MUL1, ARIH1, and SIAH1 (Figure 1A), which have been found at the OMM of damaged mitochondria, can ubiquitinate OMM proteins, which will in turn recruit the phagophore to engulf and degrade the damaged mitochondria [30]. MUL1 can act not only as a ubiquitin ligase but also as a mitophagy receptor since it contains a LIR domain for association with LC3 [30] (Figure 1B). It should be noted that these ubiquitin E3 ligases may have a different set of OMM substrates than the ones used by Parkin [30]. PINK1 activates both ARIH1 [43] and SIAH1 [44], suggesting that multiple PINK1 pathways are required to support proper mitophagy (Figure 1A).

GP78 is an ER membrane–anchored ubiquitin ligase capable of inducing mitophagy since it interacts directly with MFN1 and MFN2, and induces their ubiquitination and degradation [45].

ATF4F binds to Parkin promoter and upregulates Parkin expression [46]. Interestingly, c-Jun can also bind to the same site, but acts as a transcriptional repressor of Parkin gene expression [47]. PERK mediates the phosphorylation of EIF-2A, which, in turn, triggers gene expression via ATF4F. TFEB, a regulator of mitochondrial biogenesis, is translocated to the nucleus following mitophagy induction to maintain the autophagy–lysosome pathway and mitochondrial biogenesis [48]. Other transcription factors such as MITF and TFE3, belonging to the same family, show similar regulation during mitophagy [49]. E2F1 and SP1 bind as a complex to the promoter of MFN2 to upregulate its expression [50].

Ubiquitinated mitochondrial substrates can recruit several autophagy receptors, including SQSTM1/p62, NBR1, OPTN, NDP52, and TAX1BP1, through their ubiquitin-binding domains (Figure 1A). It should be noted that TBK1 phosphorylates protein p62 promoting mitophagy [51], and that SAMM50 interacts directly with p62/SQSTM1 in a cooperative way to mediate efficient mitophagy [38]. OPTN uses TBK1, but not ULK1, to initiate mitophagy, while NDP52 uses ULK1 [52]. TBK1 also phosphorylates OPTN [53], and recruits ATG9A, which results in the de novo formation of autophagosomes on the surface of damaged mitochondria, thus being essential for mitophagy [54]. Upon the occurrence of mitochondrial damage, RABGEF1 is recruited to mitochondria and, in turn, recruits RAB5A. Then, RAB5A recruits RAB7B via MON1B/CCZ1B complex [55]. ATG9 vesicles and LC3-labeled autophagic membranes are assembled on mitochondria in a RAB7-dependent manner [56]. TBC1D15 also inhibits RAB7 activity and associates with both the mitochondria through binding to FIS1 (located at the OMM) and to the isolation membrane by interacting with LC3 through its LIR domain, thus constraining autophagosome morphogenesis to the cargo [57].

Although the Parkin/PINK1 axis is the main regulator of mitophagy, other Parkin-independent mitophagy pathways (Figure 1B) have also been described that involve BNIP3 and its homolog NIX/BNIP3L [30]. Both are located at the OMM, allowing the exposition of their N-terminus LIR domain into the cytosol and thus facilitating binding to LC3. ULK1 phosphorylates BNIP3 and NIX, promoting their interaction with LC3 and thus mitophagy [58]. BCL-xL interacts with both NIX and BNIP3 to protect cells from NIX-BNIP3-induced death [59]. Receptor-mediated mitophagy can likely begin with the de novo generation of autophagosomes on the mitochondrial surface, as well as with the recruitment of mitochondria to pre-formed phagophores, since BNIP3 and NIX have been reported to interact with and become phosphorylated by ULK1 during mitophagy [54]. NIX is also instrumental to the rapid mitochondrial depolarization that is important for the accumulation of Pink1 on the mitochondria [60].

BNIP3 is also a transcriptional target of HIF-1, E2F1, FOXO3, RAS, and p53, while NIX is regulated by HIF-1- and p53. p53 inhibits the transcription and expression of BNIP3, resulting in mitophagy stagnation [61]. In contrast, NIX is upregulated by p53. Both BNIP3 and NIX are transcriptionally activated by HIF-1 [62]. FOXO3 also regulates mitochondrial function and integrity by binding to the *BNIP3* upstream promoter region and increasing *BNIP3* expression [63].

FUNDC1 can be positively or negatively regulated by the phosphorylation of residues near the LIR domain (Figure 1B). For instance, ULK1 can phosphorylate FUNDC1 at Ser17 to allow its association with LC3, thus promoting the activation of mitophagy. In contrast, SRC and CK2 kinases inhibit the interaction between FUNDC1 and LC3 by phosphorylating Tyr18 and Ser13, thereby inhibiting mitophagy [64]. On the other hand, PGAM5 phosphatase interacts with and dephosphorylates FUNDC1 Ser13, enhancing its interaction with LC3 and thus also inducing mitophagy. OPA1, which localizes at the inner mitochondrial membrane and intermembrane space (Figure 1B), interacts with FUNDC1, reducing mitophagy [65]. FUNDC1 can also be ubiquitinated by MARCHF5, targeting it for proteasomal degradation and thus limiting mitophagy.

AMBRA1 has a LIR motif and the capacity to induce mitophagy, with HUWE1 being a key inducing factor in AMBRA1-mediated mitophagy [66] (Figure 1B).

FKBP8 is also anchored in the OMM and has a LIR motif, and thus efficiently recruits LC3 to damaged mitochondria in a LIR-dependent manner [67]. BCL2L13 can also bind to LC3, leading to the formation of mitophagosomes [68]. MUL1, which regulates PINK1/Parkin mitophagy as a ubiquitin E3 ligase, can also interact with LC3 through a LIR motif [69].

LC3 also mediates the targeting of MTX1 and/or MTX2, both located in the OMM, possibly through one of the non-canonical LIR motifs in the unstructured cytosol-exposed regions of metaxins [70]. These proteins can also be found in association with SAMM50, and can thus be involved in cristae junction formation and/or maintenance. Metaxins can also interact with p62, which could play a role in clustering membrane-embedded piecemeal mitophagy substrates [70]. SAMM50, which can interact with PINK1 to facilitate its degradation (Figure 1A), can also bind to LC3 through the LIR motif located in its N-terminal region (Figure 1B), acting, with the involvement of the autophagy receptor p62, as a receptor for the degradation of metaxins. SAMM50 and p62 cooperate to mediate efficient mitophagy [38].

PBH2, an inner mitochondrial membrane (IMM), also uses a LIR motif to interact with LC3 (Figure 1B), thus acting as a receptor for the mitophagy machinery [71]. Access of LC3 to PHB2 requires prior disruption of the OMM, which can be achieved by the Parkin-mediated degradation of OMM proteins, leading to exposure of the PHB2 LIR domain to LC3, and subsequent mitophagy [72].

The LIR domain of VCP can also interact with LC3, and this interaction seems not to be dependent on the Parkin/PINK1 pathway (Figure 1B). VCP accumulation can lead to the excessive activation of mitophagy, thus inducing neuronal damage as in models of Huntington’s disease [73]. NLRX1 also has a LIR motif that induces binding to LC3 (Figure 1B), independently of PINK1 and Parkin [74].

At the nucleus, P53 can bind to the promoter of *FOXO3* to stimulate its transcription [75]. Moreover, FOXO3 can bind to the promoter of *BNIP3*, enhancing its transcription and promoting mitophagy.

Mitochondrial dysfunction in SCA3/MJD is strongly suggested by changes in the morphology of the mitochondria (ring-shaped/doughnut-like mitochondria), decreased cerebellar mitochondrial respiration, and ATP production in SCA3/MJD animal and cell models [21,22,23,24,25]. It seems, however, that mitophagy is not induced by the activation of the Parkin/PINK1 pathway. Ataxin-3 interacts with Parkin, and it has been shown that polyQ-expanded ataxin-3 is linked to an abnormal loss of Parkin [76]. Nevertheless, the decrease of about 80% in the Parkin levels in the brain lysates of transgenic mice expressing expanded ataxin-3 (MJD84.2), compared with wild-type [76], seems not to be due to the co-aggregation of Parkin with expanded ataxin-3 in insoluble inclusions, as no changes in Parkin levels were observed in the insoluble brain fractions of transgenic mice compared to WT animals. These observations suggest a lower expression of the *Parkin* gene, a faster clearance of the protein from cells, or both. The interaction between ataxin-3 and Parkin is direct and is greatly enhanced by Parkin self-ubiquitination [76]. It should be noted that both the WT and expanded ataxin-3 forms can deubiquitinate Parkin. Nevertheless, the expanded ataxin-3 form deubiquitinates Parkin more efficiently than the WT ataxin-3 form, and, remarkably, expanded but not WT ataxin-3 promotes the clearance of Parkin via the autophagy pathway [76]. No changes have been observed in the P(Ser65)-Parkin/Parkin proportion in the mitochondria isolated from mutant mice, or in the mitochondrial and cytosolic fractions derived from PC6-3 cells, when both are compared with the WT [22]. It should, however, be noted that, in the disease models used by Almeida et al. [22], there was no decrease in the number of mitochondrial copies either, as observed in other SCA3/MJD disease models [23,24,25].

According to the Parkin/PINK1 dependent mitophagy model, lower amounts of Parkin should lead to decreased mitophagy, but, as pointed out above, most reports do observe a decrease in the number of mitochondrial copies, suggesting increased mitophagy in SCA3/MJD. Moreover, no changes in MFN2 protein levels (a substrate of Parkin) have been observed in MJD135 mice or PC6-3 Q108 cells, thus further supporting the view that, in patients with SCA3/MJD, mitophagy is not induced by the activation of the Parkin/PINK1 pathway [22]. It should be noted that, in these disease models, a decrease in mitochondrial membrane potential has been observed, suggesting increased mitophagy [22]. Therefore, an alternative to the PINK1/Parkin mitophagy pathway is being used in patients with SCA3/MJD.

In patient-derived SCA3/MJD cells (MEF 148Q or iHF SCA3), mitophagy is suggested to occur in the presence of intact IMM, resulting in a normal cristae structure and no changes in mitochondrial membrane potential [77]. The ATP production is, however, significantly reduced and the cell viability is compromised [77]. The reduced PINK1, TOMM20, OPA1, and MFN2 protein levels, as well as the lack of changes in protein levels observed for p62 and LC3, suggests that the PINK1/Parkin mitophagy pathway is not being activated. Nevertheless, the VDAC1 (a substrate of Parkin) protein levels are increased, which leads the authors to suggest that this protein may be involved in a new mitophagy pathway that is independent of protein ubiquitination, since expanded ataxin-3 efficiently removes polyubiquitin chains from VDAC1.

It has been shown that expanded ataxin-3 downregulates the BCL-xL (another component of the mitophagy pathway) expression in cultured cerebellar, striatal, and substantia nigra neurons, which leads to the mitochondrial release of cytochrome *c*, which in turn induces apoptotic neuronal death [78]. In normal cells, BCL-xL inhibits mitochondria-mediated apoptosis and promotes cell survival by preventing the mitochondrial release of cytochrome c [79,80].

The expanded ataxin-3 induces ER stress, leading to an increase in GP78 that, in turn, promotes the ubiquitination and degradation of the expanded ataxin-3. Nevertheless, it is possible that the expanded ataxin-3 is more resistant to degradation, which would explain the abnormal accumulation of ataxin-3 aggregates and which would contribute to ER stress [81] and increased mitophagy through a Parkin/PINK1 independent pathway [45].

There are other ataxin-3 interactors, such as VCP, for which it is known that their expanded form binds them with increased affinity [82,83], but whether this leads to an increase or decrease in mitophagy has not been addressed. A decreased level of VCP, however, limits LC3 recruitment, and thus decreases autophagosome formation [84].

In tissue from patients with SCA3/MJD, as well as in transgenic mice and in the lentiviral-based rat model, Beclin1 levels are decreased [85]. Beclin1 can interact with both WT and expanded ataxin-3, but mutant ataxin-3 binds Beclin1 more strongly than the WT form [86].

TBK1, which is involved in mitophagy, is required for the direct phosphorylation of ataxin-3 [87]. Since the ataxin-3 interactor network is very large, it is still possible that other proteins involved in mitophagy have not yet been identified as ataxin-3 interactors.

In this work, we present an in silico pipeline called auto-p2docking that was used to identify putative ataxin-3 interactors (including novel interactors) out of the 68 proteins known to be involved in mitophagy (according to the KEGG database), and we use the interacting regions (IRs) reported in Sousa e Silva et al. [7] to validate the results. Since AlphaFold 3D protein predictions have, in general, a good overlap in their binding site compared to their corresponding PDB structures, we use these to perform docking analyses. For the proteins that do not present IRs, we also perform docking with WT ataxin-3 using the D-I-TASSER 3D protein structure predictions, since it has been observed that, for some proteins, AlphaFold structures present striking variations at the side-chain level within the binding site [88]. For the predicted ataxin-3 interactors, we find differences in binding affinities between expanded and WT ataxin-3 forms, indicating that, in SCA3/MJD, mitophagy is severely affected, mainly through the activation of a Parkin/PINK1 independent pathway.

## 2. Results

### 2.1. The Auto-p2docking Pipeline

**Figure 2:**
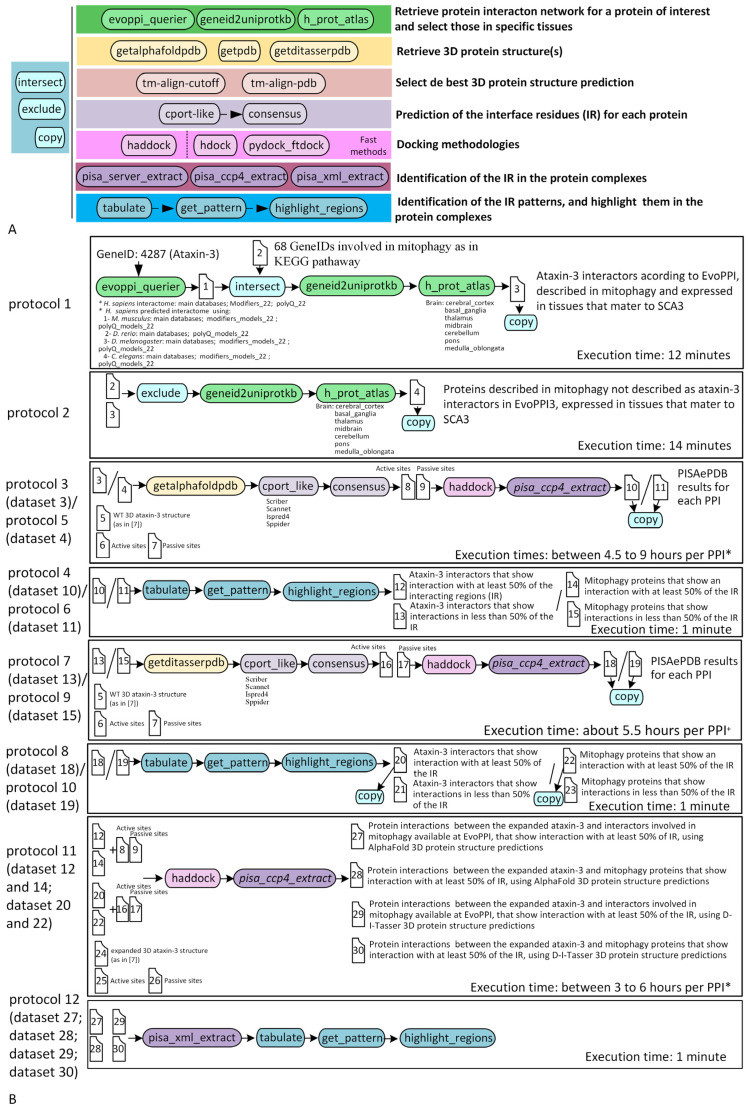
The 22 auto-p2docking pipeline modules, (**A**) grouped according to their general purpose and used to build the 12 protocols (**B**) used in this work (see Table S2 for details). * depending on the hardware specifications (see Section 4; Table S3); the estimate is for a protein with 350 amino acids. + results were obtained using a single machine; the estimate is for a protein with 350 amino acids.

Auto-p2docking is a modular and flexible pipeline with 22 modules (Figure 2A) that can be combined to build different protocols (Figure 2B), which is done by declaring the modules to be run, the name of the input folder, and that of the output folder in a pipeline file, and the absolute path, as well as, if needed, the values of the variables to be used by different programs, in a config file. It is distributed as a Docker image, and, thus, the user only needs to install Docker to use it. By using controlled program versions, by explicitly declaring the operations that have been performed as well as the variable values in the pipeline and config file, and by organizing the output files, the raw results of any project can be easily shared and reproducibility is ensured. Besides the project folder that contains all files and folders used and those generated during the analysis, a special folder, named “files_to_keep”, is created that contains the minimum amount of information that should be made available to guarantee reproducibility.

Briefly, a full pipeline implements the following steps, which can be run all together or separately in different smaller pipelines, and which are performed here for convenience. The pipeline starts with the identification of the interactors of a particular protein using the information available at EvoPPI3 (http://evoppi.i3s.up.pt/ (accessed on 20 July 2024); [89]), an aggregator of the main protein–protein interaction databases. Then, the interactors are filtered according to their expression in selected tissues using the information available at The Human Protein Atlas (https://www.proteinatlas.org/ (accessed on 20 July 2024)), as in protocol 1 (Figure 2B).

The 3D structure of the proteins of interest is then retrieved from the AlphaFold database (https://alphafold.ebi.ac.uk (accessed on 20 July 2024)), which uses deep learning techniques to predict the 3D structure directly from the amino acid sequence (as in protocol 3 and protocol 5; Figure 2B), or/and from the HPmod database (https://zhanggroup.org/HPmod/ (accessed on 20 July 2024)), which uses D-I-TASSER [90] (as in protocol 7 and protocol 9; Figure 2B), an advanced protein structure and function prediction tool that integrates a set of neural network predictors to predict spatial relationships between amino acids such as contacts, distances and hydrogen bonding patterns. In order to restrict the sampling search during the protein–protein docking approach, predictions are obtained regarding interacting residues and non-interacting residues for each protein as unbound monomer surfaces. For that, structure-based methods such as ISPRED4 (https://ispred4.biocomp.unibo.it/welcome/default/index (accessed on 20 July 2024)) and SPPIDER (http://sppider.cchmc.org/ (accessed on 20 July 2024)) [91,92] are used, as well as sequence-based methods such as SCRIBER (http://biomine.cs.vcu.edu/servers/SCRIBER/ (accessed on 20 July 2024)) and PSIVER (https://mizuguchilab.org/PSIVER/ (accessed on 20 July 2024)) [93,94], as in protocol 3, protocol 5, protocol 7, and protocol 9 (Figure 2B). The user can decide whether to select one or all models. Protein–protein docking inferences are performed using structure-based molecular docking methods (HADDOCK; [95]) to model the structural orientations of the two interacting proteins (as in protocol 3, protocol 5, protocol 7, and protocol 9; Figure 2B). Methods based on chemical thermodynamics (that depend on parameters such as the interface area, residue/atom composition and contacts, hydropathy index charge distribution, and topological complementary, among others), such as those implemented in the Protein, Interfaces, Structures, and Assemblies functions (PISAePDB [96]), are then used to identify biological relevant contacts from the other crystal contacts present in the PDB file of the protein complex (as in protocol 8, protocol 10, and protocol 12; Figure 2B). The results are tabulated and interacting regions (defined according to the user specifications) are identified and shown on the illustration of the protein–protein complex. The 12 protocols used in this work (Figure 2B and Table S2) and the entire project are available at Zenodo (https://doi.org/10.5281/zenodo.14761185).

To address running times, it must be taken into account that the “cport-like” and “haddock” modules likely take longer to process larger proteins than smaller proteins. Moreover, to speed up the analyses, three computers (C1–C3; Section 4.) with different specifications were used. In protocols 3, 5, 7, and 9, “cport-like”, “haddock”, and “pisa-ccp4-extract” modules are used (Figure 2B). Nevertheless, for running time considerations, we analyzed protocols 3 and 5 together and protocols 7 and 9 together, since the running times could be affected by the 3D protein structure used (Figure 2B). A linear correlation between the protein size and running time was obtained for protocols 3 and 5 and protocols 7 and 9 in the different computers (Table S3). In the following formulas, y is the estimated running time (in minutes) and x the protein size (number of amino acids). The prediction used is for a protein with 350 amino acids, about the average size of a protein. The results for protocols 3 and 5 (for C1 (N = 8) y = 1.092 × x + 159.920, Pearson’s R = 0.572, *p* = 0.139, prediction = 542 min; for C2 (N = 30) y = 0.522 × x + 158.669, Pearson’s R = 0.816, *p* < 0.001, prediction = 341 min; for C3 (N = 20) y = 0.438 × x + 108.679, Pearson’s R = 0.78, *p* < 0.001, prediction = 262 min) imply a correlation between the protein size and running time (with the exception of protocols 3 and 5 (that were analyzed together) in C1, for which the correlation is non-significant, but this could be due to the small number of data points being considered). There is also an impact of the machine used, since C3 and C2 are expected to produce results in 48.3% and 62.9% of the time needed for C1, respectively. The results for protocols 7 and 9 were all obtained using C1 (N = 38) y= 0.593 × x + 124.087, Pearson’s R = 0.716, *p* < 0.001, prediction = 332 min, and again imply a linear correlation between the protein size and running time. The comparison of these results with those given above for protocols 3 and 5 in C1 suggests that it takes less time to analyze D-I-TASSER than AlphaFold structures, but since the “cport-like” module is performed using public servers, it is also conceivable that the time difference between the two conditions could be due to how busy the public servers are on a given day. The results for protocol 11 (for C1 (N = 11) y = 1.091 × x − 0.668, Pearson’s R = 0.739, *p* < 0.01, prediction = 381 min; for C2 (N = 21) y = 0.536 × x + 108.102, Pearson’s R = 0.753, *p* < 0.001, prediction = 296 min; for C3 (N = 13) y = 0.248 × x + 101.164, Pearson’s R = 0.652, *p* < 0.05, prediction = 188 min) also show a correlation between the protein size and running time, and that the running times are affected by the specifications of the machine used. Since, in this case, the “cport-like” module is not used, this reflects the running time of the “haddock” module (the time it takes to run PISAePDB is negligible). C3 and C2 are expected to produce their results in 77.7% and 49.3% of the time needed for C1, respectively. The higher clock speed of C3 (1.9 times faster) when compared with C2 likely accounts for the difference in execution time between C3 and C2 (1.6 times slower).

### 2.2. Mitophagy Pathway Proteins That Are Ataxin-3 Interactors

According to KEGG pathways, there are 104 proteins that define 68 groups involved in animal mitophagy, which are defined according to their functions (Table S1). For each group we have selected one protein only, since, in all cases (N = 17), except for Parkin substrates, the different genes belong to the same gene family (Table S1). Regarding Parkin substrates, we have analyzed three proteins (MFN2, FKBP8, and TOMM70), although only MFN2 is commonly reported as a Parkin substrate (see Introduction). Of the 68 selected proteins (Table S1), according to EvoPPI3, there are 32 interactors that have been described as ataxin-3 interactors or modifiers of ataxin-3, when considering human main databases (N = 17), human SCA3/MJD mutant cell lines (N = 12; 11 are in common with those reported in main databases), *Danio rerio* SCA3/MJD mutants (N = 10; three in common with those in human main database), *Mus musculus* modifier screens (N = 6; two in common with those in human main database), *Drosophila melanogaster* modifier screens (N = 11; three in common with those in human main database), the *Caenorhabditis elegans* main database (N = 1), and *C. elegans* modifier screen (N = 1); Table 1; see the results of protocol 1. All of these proteins are, according to the Human Protein Atlas, expressed in tissues that matter in SCA3/MJD [97], namely the cerebral cortex, basal ganglia, thalamus, midbrain, pons, medulla oblonga, and cerebellum (protocol 1). It should be noted, however, that most of the reported PPIs have been obtained using high-throughput approaches, and, thus, some of them could be false ataxin-3 interactions [89]. Being present in databases and showing the expected pattern of interaction at the IR [7] means that they are true positives. Of the 68 mitophagy-related proteins in the KEGG database, for 36 there is no evidence that they could be ataxin-3 interactors, but since the experimental approaches used to obtain PPIs do not take in account transient dynamics, post-translational modifications, proteins with disordered regions, or physiological conditions [98], and since these proteins are expressed in tissues that are important in SCA3/MJD (protocol 2), they could be ataxin-3 interactors that are yet to be described. Therefore, we have performed protein–protein docking analyses for the two groups separately.

For 27 (84.4%) out of the 32 putative ataxin-3 interactors listed in EvoPPI3, a docking solution was obtained when using the WT ataxin-3 structure used in [7] (and the same active and passive sites), and the AlphaFold predicted 3D protein structures for these ligands. Out of these 27 interactors for which a docking solution was obtained, 14 (51.9%) are considered true interactors, since the docking solution shows that more than 50% of the residues of the five interacting regions (IRs) described in [7] are in interface regions (Table 2). Since proteins do not fold into a single conformation, but instead are able to “flip” between different conformations to be functional [99], and since small variations in 3D protein structures can have a large impact on the results obtained in molecular docking [88], for the remaining 13 putative ataxin-3 interactors, as well as for four out of five interactors for which no docking solution was obtained (for HUWE1 (Q7Z6Z7; 10075) no structure is available at the HPmod database), the analyses were repeated using, this time, the D-I-TASSER 3D structure predictions available at the HPmod database. For 7 (41.2%) out of the 17 putative ataxin-3 interactors, the use of a different predicted 3D structure revealed that they are also true ataxin-3 interactors when using the same criteria as above (Table 3). Therefore, when performing this kind of analysis, more than one 3D protein structure should be used.

The 10 putative ataxin-3 interactors listed in EvoPPI3 for which we found nothing in our docking analyses to support that they are true ataxin-3 interactors (assuming the subjective criteria that at least 50% of the ataxin-3 residues in the five IRs defined in [7] should be interfacing residues) are supported by the following evidence: TRAF2 (Q12933; 5970) is reported in the human main databases, human-polyQ, and *D. melanogaster* predicted modifiers; NFKB (Q04206; 5970), LC3 (O95166; 11337), and SMURF1 (Q9HCE7; 57154) are reported in human main databases and human-polyQ; p62 (Q13501; 8878) is reported in human main databases; EIF-2A(P05198; 1965), OPA1 (O60313; 4976), and RAB5A (P20339; 5868) are reported in the *D. rerio* predicted polyQ database; USP8 (P40818; 9101) and FKBP8 (Q14318; 23770) are reported in *D. melanogaster* predicted modifiers. Given this evidence (5 out of 10 are reported in human main databases), it is likely that the criterion of 50% interaction at the IRs is too conservative. Nevertheless, even considering a value of 40% at the IRs, proteins such as TRAF2 (Q12933; 5970), reported in three different databases as being an ataxin-3 interactor, are not supported in our inferences. The use of additional 3D protein structure predictions, or detailed studies on TRAF2, may help solve this issue.

When looking at the 36 proteins in the KEGG database that are not reported as ataxin-3 interactors in EvoPPI3, when using AlphaFold 3D protein predictions, we obtained no docking solution for five interactors (OPTN (Q96CV9; 10133); BCL2L13 (Q9BXK5; 23786); NIX (O60238; 665); ATG9B (Q674R7; 285973); and TFEB (P19484; 7942)), and we found 11 (35.5%) out of 31 to be likely ataxin-3 interactors, since, for these, more than 50% of the ataxin-3 residues in the IR defined in [7] are interfacing residues (Table 4). The docking analyses were repeated for the remaining 20 proteins plus the five for which no docking solution was obtained, but this time using the structures available at the HPmod database. For four proteins (ULK1 (O75385; 8408); ATF4F (P18848; 468); USP15 (Q9Y4E8; 9958); TBC1D15 (Q8TC07; 64786)), no docking solution was found. For 13 (61.9%) out of the remaining 21 proteins, we obtained evidence that they are ataxin-3 interactors (Table 5). Therefore, by performing docking analyses, here, we identify 24 (66.7%) proteins out of 36 proteins that are involved in mitophagy and that are very likely ataxin-3 interactors, although there is no such report in the literature yet.

In summary, even when using what is likely a conservative approach, out of the 68 proteins studied here that are involved in mitophagy, we found evidence that 45 (66.2%) are ataxin-3 interactors (21 already reported in EvoPPI3 as ataxin-3 interactors).

### 2.3. Ataxin-3 Interactors That Belong to the Mitophagy Pathway and Are Predicted to Have Different Binding Affinities with the WT and Expanded Forms

Of the 45 ataxin-3 interactors involved in mitophagy that are identified herein, 26 show a larger number of interfacing residues with the expanded ataxin-3 form compared with the WT ataxin-3 form. Of these, 15 show an increase in the percentage of interfacing sites larger than 10% (Table 6). On the other hand, 16 ataxin-3 interactors show a smaller number of interfacing residues with the expanded ataxin-3 form compared with the WT form. Of these, 11 show a decrease in the number of interfacing residues below 10% (Table 6). Ataxin-3 interactors that show an increase or decrease larger than 10% in their percentage of interfacing sites with the expanded ataxin-3 form compared with the WT form are likely relevant in SCA3/MJD. Three (Beclin1 (Q14457; 8678), GP78 (Q9UKV5; 267), and MARCHF5 (Q9NX47; 54708)) out of the four proteins involved in mitophagy that have been experimentally shown to bind more strongly to the expanded ataxin-3 form than the WT form [20,81,86,100] are in the list of proteins predicted to show an increase in their percentage of interfacing sites larger than 10% with the expanded ataxin-3 form when compared with the WT form. Although VCP (P55072; 7415) shows an increase in the predicted percentage of interfacing sites with the expanded ataxin-3 form compared with the WT form, the difference is only 5.6%. Although our predictions fit what is reported in the literature, these results and the other predictions made here regarding binding differences between WT and expanded ataxin-3 forms must be validated using methodologies that, ideally, take into account the presence of protein aggregates.

### 2.4. Predictions Regarding Mitophagy in SCA3/MJD

**Figure 3:**
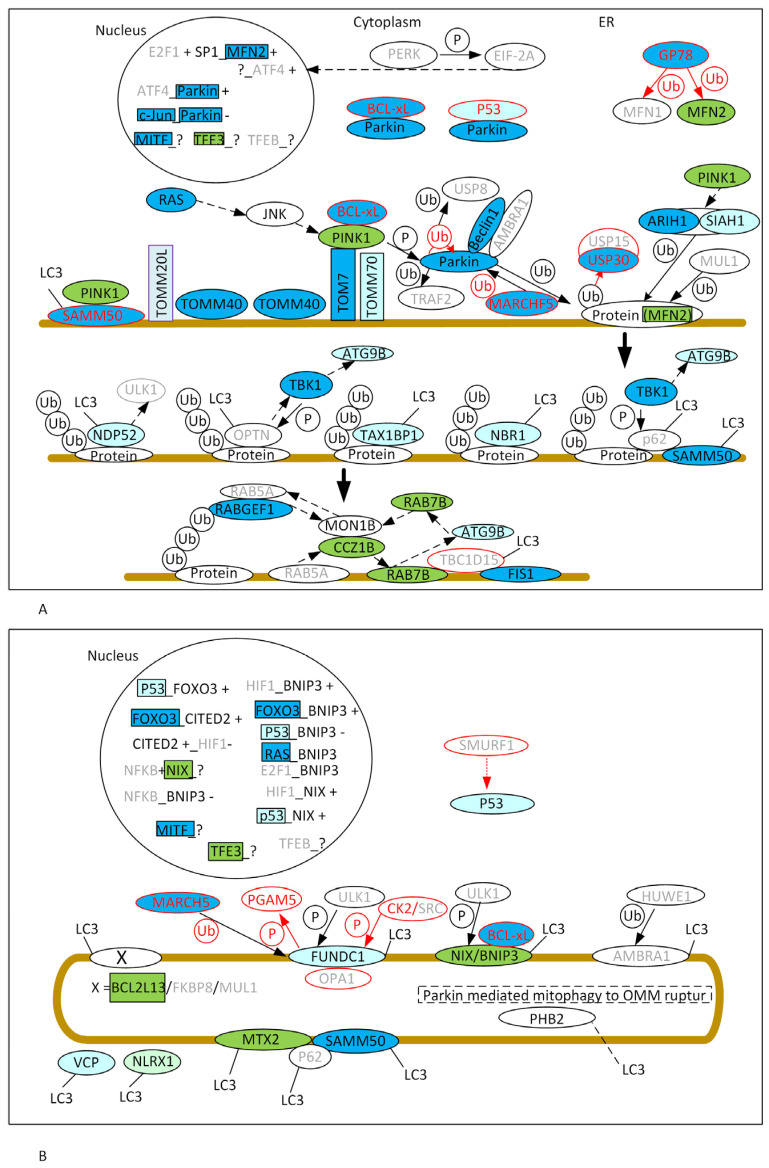
PPI in PINK1/Parkin-dependent (**A**) and -independent (**B**) mitophagy in animals (see Introduction) for the 68 mitophagy proteins, according to KEGG pathways. Proteins labeled in red are inhibitors, and those in grey are the proteins for which there is no evidence that they interact with ataxin-3. Ub stands for ubiquitination, and P for phosphorylation. The mitochondria is marked with a brown line. Proteins highlighted in blue are those predicted to show increases of 10% (dark) and 5% (light) in the percentage of interfacing residues with the expanded ataxin-3 form and with the WT form. Proteins highlighted in green are the proteins predicted to show decreases of 10% (dark) and 5% (light) in the percentage of interfacing residues with the expanded ataxin-3 form and with the WT form. Arrows represent PPIs, in red are negative interactions and in black positive interactions. In the nucleus, the first name before the underscore is the name of the transcription factor and, after the underscore, the name of the gene being regulated by the transcription factor and the signal at the end (₊ or ₋) shows whether the gene is up or downregulated, respectively, when the transcription factor binds the promoter of the specified gene.

We assume that a protein that is predicted to show greater affinity with the expanded ataxin-3 form than with the WT form will present decreased protein levels. On the other hand, when a protein is predicted to show a stronger interaction with the WT ataxin-3 form than with the expanded form, we assume that its levels are increased. Under these assumptions, our data suggest that the PINK1/Parkin pathway is severely altered, since most of the proteins are predicted to bind differently with the expanded ataxin-3 form when compared with the WT form (Figure 3A). For some proteins, it is, however, difficult to infer their protein levels. For instance, for PINK1, we expect high protein levels in cytoplasm, since we predict a weaker interaction with the expanded ataxin-3 form compared with the WT form. Moreover, SAMM50 is also predicted to be present in lower amounts in SCA3/MJD (depletion of SAMM50 results in PINK1 accumulation [37]), and, thus, we could expect higher PINK1 levels at the OMM. Nevertheless, we also predict a lower amount of TOM7, and PINK1 forms a high-molecular-weight complex with the TOMM machinery, which seems to be needed to accumulate PINK1 at the OMM [101]. Therefore, we have no clear prediction for PINK1 protein levels at the OMM (Figure 3A). Moreover, lower PINK1 protein levels at the OMM do not necessarily imply a lower activation of the PINK1/Parkin pathway, since BCL-xL levels are also predicted to be reduced, and, thus, there would be more PINK1 available to phosphorylate Parkin [42]. RRAS2, which is also predicted to show reduced levels, may also have an impact on PINK1 levels, although in this case it is difficult to make clear predictions since the details of this mechanism are unknown.

For Parkin, we expect lower protein levels due to interaction differences between the expanded ataxin-3 form and the WT form (Figure 3A). Moreover, since Beclin1, which interacts with Parkin to ensure its efficient delivery to dysfunctional mitochondria [31], is also inferred to have reduced protein levels, we expect that there is not that much Parkin at the OMM. Nevertheless, BCL-xL, MARCHF5, and c-Jun, which are also inferred to show reduced protein levels, negatively regulate Parkin (BCL-xL binds to Parkin, forming oligomers in cytoplasm and preventing the recruitment of Parkin to the OMM [42]; MARCHF5 binds to Parkin, promoting Parkin degradation; c-Jun binds to the promotor of the Parkin gene, reducing its expression), thus contributing to higher Parkin levels. Without knowing the relative effect of these proteins, it is difficult to make a prediction about Parkin levels at the OMM.

Regarding the amount of MFN2 substrate (Figure 3A), GP78 ubiquitinates MFN2, leading to its degradation, and since we predict lower GP78 levels in SCA3/MJD, there should be an increase in the MFN2 levels. Moreover, WT ataxin-3 shows a stronger association with MFN2 than with the expanded ataxin-3, which is also predicted to lead to increased MFN2 levels.

The MUL1 levels are predicted not to be affected by the presence of a polyQ expansion in ataxin-3, and, thus, could, in principle, perform MFN2 ubiquitination. Moreover, for USP30, we predict reduced levels. Since USP30 acts as a mitophagy brake (by removing ubiquitin chains from the substrate), we predict higher levels of ubiquitinated MFN2. Although, for ARIH1, we predict reduced protein levels in SCA3/MJD due to binding differences with the two ataxin-3 forms, higher PINK1 levels could, however, compensate for this decrease by indirectly promoting its increase (Figure 3A). The same could be happening, to a lower extent, with SIAH1. The autophagy receptor (LC3 adaptors) proteins seem not to be strongly affected by the polyQ expansion, and, thus, in SCA3/MJD, Parkin independent but dependent on ubiquitinated proteins at the OMM mitophagy may not be compromised (Figure 3A). Nevertheless, TBK1, required by the autophagy receptors p62 and OPTN to initiate mitophagy, as well as for the de novo formation of autophagosomes on the surface of damaged mitochondria [54], is predicted to show reduced levels in SCA3/MJD. Furthermore, RABGEF1 is recruited to mitochondria to recruit RAB5A to initiate autophagosome formation, and RABGEF1 is also a protein that is predicted to have decreased levels in SCA3/MJD. Nevertheless, we also predict increases in RAB7B (not just because of binding differences with the two ataxin-3 forms, but also because of the predicted decrease in its inhibitor, TBC1D15-FIS1) and CCZ1B. Both are needed for the assembly of LC3-labeled autophagic membranes on mitochondria [56], and could thus compensate for the predicted lower levels of TBK1 and RABGEF1.

BNIP3 and NIX can trigger mitochondrial depolarization, thus causing mitophagy [102]. For these two proteins, we expect higher levels in SCA3/MJD (Figure 3B). For BCL2L13, which also promotes mitophagy [102], we also predict an increase in protein levels in SCA3/MJD using the same rational (Figure 3B).

For metaxins (herein we studied MTX2, which is located at the OMM and faces the cytosolic compartment through direct interaction with its partner MTX1), we also expect high protein levels in SCA3/MJD, not just because we predict a stronger interaction with the WT ataxin-3 form compared with the expanded form, but also because the levels of SAMM50 are also reduced. Overall, our predictions point to an increase in Parkin-independent mitophagy in SCA3/MJD (Figure 3B).

## 3. Discussion

The auto-p2docking pipeline is an innovative modular approach to PPI prediction that overcomes the limitations of manually performing multiple in silico approaches. Containerized in Docker, auto-p2docking ensures reproducibility and reduces errors through a simplified configuration and system portability. Here, we present 12 protocols, based on 22 modules (Figure 2A), that allow us to compare the results reported in the different databases available in EvoPPI3 (main databases, predicted interactors, polyQ_22, and Modifiers_22; [89]), to select a list of interactors for a given protein, to check the expression of the genes encoding these proteins in the tissues of interest to the user (according to The Human Protein Atlas), to obtain 3D protein structures from different databases (namely AlphaFold and HPmod), to predict active and passive residues for each protein, to apply docking methodologies such as HADDOCK, to identify and select the best docking solution, to explore macromolecular interfaces, and to identify interacting regions according to user specifications and visualize them in the protein complex (Figure 2B). These modules can be specified according to user’s needs, creating other protocols. The key features include intermediate file storage for traceability, automated error checking, a user manual, and graphical pipeline visualization. By automating manual and error-prone processes, auto-p2docking significantly reduces the analysis time required and improves accuracy. To take advantage of computational resources, the list of proteins to be studied can be analyzed in different runs and then joined for the final steps of the analysis, as performed herein.

Using auto-p2docking, we identify 45 (66%) out of the 68 proteins that are assigned as involved in mitophagy according to the KEGG database as ataxin-3 interactors, using the conservative assumption that, in order to be a true ataxin-3 interactor, at least 50% of the amino acid residues identified in the IR [7] should be present in interfacing regions. Of these, only 21 have been reported as ataxin-3 interactors, and only 11 (52%) are reported in human main databases; seven are also reported in human SCA3/MJD mutant cell lines and two are reported in the *D. melanogaster* modifier screens database (Table 1). Therefore, data on other SCA3/MJD model systems can be used to complete the ataxin-3 interactome network. Fifty three percent of the predicted ataxin-3 interactors are novel, thus reinforcing the usefulness of in silico methodologies to complete protein networks (Table 2, Table 3, Table 4 and Table 5).

Of the predicted ataxin-3 interactors here reported, 15 show an increase larger than 10% in the number of interfacing sites with the expanded ataxin-3 form when compared with the WT form (Table 6). The available experimental data support our inferences, since Beclin1, GP78, MARCHF5, and VCP have been experimentally shown to have a greater affinity towards the expanded ataxin-3 form compared to the WT form [20,81,86,100], and three of them (Beclin1, GP78, and MARCHF5) are in our list of proteins that are predicted to show an increase larger than 10% in the percentage of interfacing sites with the expanded ataxin-3 form when compared with the WT form. For VCP, the increase is of 5.6%. Therefore, in silico analyses are a powerful tool to predict binding differences with the two ataxin-3 forms, and, thus, to identify ataxin-3 interactors that play an important role in SCA3/MJD.

Mitochondrial dysfunction has been demonstrated in SCA3/MJD patients, cell lines, and animal models [19,20,21,22]. Although this is a relevant feature in SCA3/MJD, it is likely that other pathways are equally affected, since there is no difference (Fisher exact test; *p* > 0.5) in the proportion of proteins that are involved in mitophagy and are predicted to show a stronger binding towards the expanded ataxin-3 form when compared with the WT form and that observed in a random sample of ataxin-3 interactors [7].

Our predictions are in agreement with previous experimental findings suggesting that, in SCA3/MJD, the observed mitophagy is mostly Parkin-independent [76,77]. We predict that most of the components of PINK1/Parkin-dependent mitophagy are affected by the presence of an expanded polyQ at ataxin-3 (Figure 3A). Furthermore, we predict that high levels of NIX, BNIP3, BCL2L13, and metaxins (such MTX2), which are LC3 receptors located on the OMM that directly bind to LC3 and recruit damaged mitochondria to autophagosomes, lead to increased PINK1/Parkin-independent mitophagy (Figure 3B), as suggested in SCA3/MJD. Although BNIP3- or NIX-induced mitophagy is associated with hypoxia [59], it is known that ataxin-3 can induce hypoxia in tumor cells [103]; thus, hypoxia-mediated mitophagy may be relevant in SCA3/MJD.

In conclusion, in silico protein–protein docking analyses can be used to support the described ataxin-3 interactors as true interactors, predict novel ataxin-3 interactors, predict interactor binding differences with the WT and expanded ataxin-3 forms, predict which proteins will show altered levels, and make predictions regarding the type of mitophagy observed in SCA3/MJD. Biochemical experiments for the validation of these predictions should now be performed. A similar approach can be used to make inferences for other neurological diseases.

## 4. Materials and Methods

The proteins involved in mitophagy were obtained from KEGG pathway database (https://www.genome.jp/kegg/pathway.html (accessed on 15 July 2024)). The WT and expanded ataxin-3 protein structure predictions, used in the in silico analyses, as well as their active and passive sites, are those reported in [7].

Auto-p2docking is a software application aimed at facilitating protein–protein docking analyses without compromising the needed flexibility required for different usages (the software is at http://bdip.i3s.up.pt/container/auto-p2docking, accessed on 15 July 2024 version 1.0.0, and the manual is at http://evolution6.i3s.up.pt/static/auto-p2docking/docs/ accessed on 15 July 2024). It also facilitates the record keeping of the program versions and the details of the performed analyses, thus contributing to reproducibility. It is mainly written in Bash and Python and is distributed as a Docker image, meaning that similar inferences can be easily made by other researchers. It is composed of 22 modules (Figure 2A) that can be combined to develop pipelines according to user needs (Figure 2B). There are some general purpose modules such as “intersect”, “exclude”, and “copy” that conduct simple operations on files, and more specific modules dedicated to well-defined tasks such as “evoppi_querier”, “geneid2uniprotkb”, “human_prot_atlas”, “getalphafoldpdb”, “getditasserpdb”, “getPDB”, “tm-align-cutoff”, “tm-align-pdb”, “cport_like”, “consensus”, “haddock”, “pisa_ccp4_extract”, “pisa_server_extract”, “pisa_xml_extract”, “tabulate”, “get_pattern”, and “highlight_regions”, used herein to develop 12 protocols in order to address the role of mitophagy in SCA3/MJD (Figure 2B; Table S2). It should be noted that structural-based methods, such as HADDOCK, are among the most accurate computational methods that can be used to make inferences on protein interactions and identify potential interaction sites on protein surfaces. Some of the available modules (such as “haddock”, and, to a lesser extent, “cport-like”), implement methods that take a long time to run. Nevertheless, when running auto-p2docking, this is not an issue, since the pipeline runs in an entirely unsupervised way. To take advantage of the available computational resources (computer 1 (C1) has an Intel^®^ (Intel, Santa Clara, CA, USA) Xeon^®^ CPU ES-2695 v4 @2.10 GHz processor (72 CPUs) and 256 GB of RAM memory, computer 2 (C2) has an AMD^®^ (Advanced Micro Devices, Inc., Santa Clara, CA, USA) EPYC^®^ 7401 24-Core @1.2 GHz processor (96 CPUs) and 1024 GB of RAM, and computer 3 (C3) has an AMD^®^ EPYC^®^ 7642 48-Core @2.3 GHz processor (192 CPUs), and 1024 GB of RAM), different protein sets are analyzed in different runs.

Typically (but not always), each module uses a Docker image that is available through the pegi3s Bioinformatics Docker Images Project (http://bdip.i3s.up.pt/ (accessed on 15 July 2024)) [104] to perform a given task, thus reducing issues related to software dependencies and configurations. Docker image versions and program versions are fully controlled, to ensure consistent and reproducible results.

Certain modules require specific variables that are specified in a unique configuration file (named config), to facilitate record keeping of the details of the analyses. These variables are prefixed with the module name, such as “haddock_all_files” for the “haddock” module, so it is easier to see where the variable value is used. Nevertheless, for the modules “evoppi_querier” and “cport_like”, variables are stored in individual configuration files to allow greater flexibility. A file called “pipeline” shows the order by which the modules should be run by declaring in each line the “module_name”, “input_folder”, and “output_folder”. Although it is not necessarily true, as branched pipelines are allowed, most of the time the output files from one module are used as the input for the next.

To prevent users from running the pipeline with errors, a feature has been added to check that the module names in the pipeline file are correct. In addition, a module called “pipeline_drawing” generates a diagram of the pipeline, allowing users to visually inspect and ensure the correct structure before execution. The pipeline diagram is in SVG format, making it easy to modify and use for visual support in scientific articles.

A feature called “block” has been added to some modules, allowing users to complete the analysis one ligand at a time, rather than processing each step for all ligands simultaneously. This feature works with a number such as “block 3”, which means that the module associated with the block instruction, as well as the next two, will all be run for one ligand at a time, allowing results to be seen more quickly for each ligand.

During pipeline execution, a folder (here referred to as “project”, but whose name is defined by the user in the config file) is created to store intermediate files from each analysis step, allowing users to trace how results were generated. Another folder called “files_to_keep” is created, which contains the minimum information that is required to reproduce the analysis under the same conditions. Nevertheless, we encourage researchers to provide both the “files_to_keep” and the “project” folder as supplementary material when publishing their results.

Finally, in the “files_to_keep” folder, a csv file is generated listing all versions of the programs and Docker images used in the analysis, along with the relevant references for these programs, making it easier to reproduce the analysis and providing valuable information that should be included in the resulting publication.

The whole project (including the input data, the “files_to_keep”, and the “project” folder) is available at Zenodo (https://doi.org/10.5281/zenodo.14761185).

## Figures and Tables

**Table 1:** EvoPPI databases supporting the interaction with ataxin-3 for 32 mitophagy proteins according to KEGG database.

EvoPPI Database	Gene Symbol (UniProtID; GeneID)
*H. sapiens* main databases; *H. sapiens* polyQ; *D. rerio* predicted polyQ; *M. musculus* predicted modifiers; *C. elegans* main database	VCP (P55072; 7415)
*H. sapiens* main databases; *H. sapiens* polyQ; *D. rerio* predicted polyQ; *M. musculus* predicted modifiers	PHB2 (Q99623; 11331)
*H. sapiens* polyQ; *D. rerio* predicted polyQ; *M. musculus* predicted modifiers; *D. melanogaster* predicted modifiers	UBC (RPS27A) (P62979; 6233)
*H. sapiens* main databases; *H. sapiens* polyQ; *D. melanogaster* predicted modifiers	TRAF2 (Q12933; 7186); Beclin1 (Q14457; 8678)
*H. sapiens* main databases; *H. sapiens* polyQ; *D. rerio* predicted polyQ	HUWE1 (Q7Z6Z7; 10075)
*H. sapiens* main databases; *H. sapiens* polyQ	Parkin (O60260; 5071); NFKB (Q04206; 5970); P53 (P04637; 7157); LC3 (GABARAP (O95166; 11337)); MARCHF5 (Q9NX47; 54708); PGAM5 (Q96HS1; 192111)
*H. sapiens* main databases; *D. melanogaster* predicted modifiers	MFN2 (O95140; 9927); TOMM20L (Q6UXN7; 387990)
*H. sapiens* main databases	GP78 (Q9UKV5; 267); BCL-xL (Q07817; 598); p62 (Q13501; 8878); SMURF1 (Q9HCE7; 57154)
*D. rerio* predicted polyQ; *M. musculus* predicted modifiers; *D. melanogaster* predicted modifiers	CK2 (CSNK2A1) (P68400; 1457)
*D. rerio* predict polyQ	EIF-2A (P05198; 1965); OPA1 (O60313; 4976); RAB5A (P20339; 5868), TOMM70 (O94826; 9868); RAS (RRAS2) (P62070; 22800)
*M. musculus* predicted modifiers	SAMM50 (Q9Y512; 25813); FIS1 (Q9Y3D6; 51024)
*D. melanogaster* predicted modifiers	c-Jun (P05412; 3725); SP1 (P08047; 6667); USP8 (P40818; 9101); FKBP8 (Q14318; 23770); FUNDC1 (Q8IVP5; 139341)
*C. elegans* predicted modifiers	FOXO3 (O43524; 2309)

**Table 2:** Docking results of the WT ataxin-3 using I-TASSER 3D predicted structure and the 32 interactors for which there is evidence of being true ataxin-3 interactors, using AlphaFold 3D predicted structures.

Gene Symbol (UniProtID; GeneID)	% of Interfacing Sites at the Five IR (N = 61)	Total Number of Interfacing Sites (N = 361)
MARCHF5 (Q9NX47; 54708)	72.131 (44)	59
MFN2 (O95140; 9927)	70.492 (43)	61
VCP (P55072; 7415)	67.213 (41)	67
FUNDC1 (Q8IVP5; 139341)	67.213 (41)	54
Parkin (O60260; 5071)	65.574 (40)	49
RAS (P62070; 22800)	60.656 (37)	42
TOMM70 (O94826; 9868)	55.738 (34)	50
SP1 (P08047; 6667)	55.738 (34)	62
CK2 (P68400; 1457)	54.098 (33)	59
PGAM5 (Q96HS1; 192111)	54.098 (33)	58
P53 (P04637; 7157)	52.459 (32)	60
Beclin1 (Q14457; 8678)	52.459 (32)	56
GP78 (Q9UKV5; 267)	52.459 (32)	51
SAMM50 (Q9Y512; 25813)	52.459 (32)	50
UBC (P62979; 6233)	49.180 (30)	35
TOMM20L (Q6UXN7; 387990)	47.541 (29)	35
LC3 (O95166; 11337)	45.902 (28)	38
BCL-xL (Q07817; 598)	45.902 (28)	75
USP8 (P40818; 9101)	42.623 (26)	59
SMURF1 (Q9HCE7; 57154)	37.705 (23)	54
p62 (Q13501; 8878)	36.066 (22)	70
OPA1 (O60313; 4976)	34.426 (21)	47
FOXO3 (O43524; 2309)	26.230 (16)	57
EIF-2A (P05198; 1965)	26.223 (16)	44
PHB2 (Q99623; 11331)	14.754 (9)	49
FIS1 (Q9Y3D6; 51024)	13.115 (8)	45
RAB5A (P20339; 5868)	11.475 (7)	50

**Table 3:** Docking results for the WT ataxin-3 using I-TASSER 3D predicted structure and the 17 interactors, using D-I-TASSER 3D predicted structures, that do not show more than 50% of ataxin-3 IR residues in interfacing regions when using AlphaFold 3D protein structure predictions.

Gene Symbol (UniProtID; GeneID)	% of Interfacing Sites at the Five IRs (N = 61)	Total Number of Interfacing Sites (N = 361)
FIS1 (Q9Y3D6; 51024)	63.934 (39)	43
PHB2 (Q99623; 11331)	60.656 (37)	57
UBC (P62979; 6233)	57.377 (35)	48
FOXO3 (O43524; 2309)	55.738 (34)	41
TOMM20L (Q6UXN7; 387990)	52.459 (32)	48
C-JUN (P05412 #; 3725)	52.459 (32)	64
BCL-xL (Q07817; 598)	50.820 (31)	53
EIF-2A (P05198; 1965)	49.180 (30)	54
p62 (Q13501; 8878)	49.180 (30)	42
LC3 (O95166; 11337)	45.902 (28)	39
FKBP8 (Q14318 #; 23770)	45.902 (28)	56
OPA1 (O60313; 4976)	44.262 (27)	57
NFKB (Q04206 #; 5970)	42.623 (26)	61
TRAF2 (Q12933 #; 7186)	36.066 (22)	46
USP8 (P40818; 9101)	26.230 (16)	38
SMURF1 (Q9HCE7; 8878)	18.033 (11)	31
RAB5A (P20339; 5868)	16.393 (10)	43

# ataxin-3 interactors for which docking using AlphaFold 3D protein structure predictions was not successful.

**Table 4:** Docking results of the WT ataxin-3 I-TASSER 3D predicted structure and the 36 interactors for which there is no evidence of ataxin-3 interaction, using AlphaFold 3D predicted structures.

Gene Symbol (UniProtID; GeneID)	% of Interfacing Sites at the Five IRs (N = 61)	Total Number of Interfacing Sites (N = 361)
CCZ1 (P86790; 221960)	72.131 (44)	53
PINK1 (Q9BXM7; 65018)	70.492 (43)	64
TBK1 (Q9UHD2; 29110)	70.492 (43)	52
RAB7B (Q96AH8; 338382)	62.295 (38)	50
TOMM40 (O96008; 10452)	57.377 (35)	50
TFE3 (P19532; 7030)	57.377 (35)	78
MON1B (Q7L1V2; 22879)	57.377 (35)	56
CITED2 (Q99967; 10370)	57.377 (35)	66
NLRX1 (Q86UT6; 79671)	54.098 (33)	36
NDP52 (Q13137; 10241)	50.820 (31)	57
NBR1 (Q14596; 4077)	50.820 (31)	54
RABGEF1 (Q9UJ41; 27342)	49.180 (30)	45
ATF4F (P18848; 468)	47.541 (29)	72
HIF1 (Q16665; 3091)	44.262 (27)	66
USP15 (Q9Y4E8; 9958)	44.262 (27)	52
SIAH1 (Q8IUQ4; 6477)	42.623 (26)	62
ULK1 (O75385; 8408)	40.984 (25)	58
BNIP3 (Q12983; 664)	40.984 (25)	66
TAX1BP1 (Q86VP1; 8887)	40.984 (25)	69
ARIH1 (Q9Y4X5; 25820)	40.984 (25)	59
TOMM7 (Q9P0U1; 54543)	39.344 (24)	44
AMBRA1 (Q9C0C7; 55626)	37.705 (23)	49
MITF (O75030; 4286)	36.066 (22)	49
SRC (P12931; 6714)	36.066 (22)	50
TBC1D15 (Q8TC07; 64786)	36.066 (22)	48
JNK (P45983; 5599)	22.951 (14)	57
E2F1 (Q01094; 1869)	21.311 (13)	74
MUL1 (Q969V5; 79594)	21.311 (13)	43
PERK (Q9NZJ5; 9451)	19.672 (12)	55
MTX2 (O75431; 10651)	16.393 (10)	45
USP30 (Q70CQ3; 84749)	14.754 (9)	69

**Table 5:** Docking results of the WT ataxin-3 I-TASSER 3D predicted structure and the 21 interactors for which there is no evidence of being ataxin-3 interactors, using D-I-TASSER 3D predicted structures.

Gene Symbol (UniProtID; GeneID)	% of Interfacing Sites at the Five IRs (N = 61)	Total Number of Interfacing Sites (N = 361)
BNIP3 (Q12983; 664)	77.049 (47)	60
ATG9B (Q674R7 #; 285973)	72.131 (44)	68
SIAH1 (Q8IUQ4; 6477)	68.852 (42)	55
NIX (O60238 #; 665)	68.852 (42)	57
MTX2 (O75431; 10651)	67.213 (41)	54
MITF (O75030; 4286)	65.574 (40)	59
RABGEF1 (Q9UJ41; 27342)	65.574 (40)	50
JNK (P45983; 5599)	62.295 (38)	50
ARIH1 (Q9Y4X5; 25820)	62.295 (38)	60
USP30 (Q70CQ3; 84749)	55.738 (34)	57
BCL2L13 (Q9BXK5 #; 23786)	55.738 (34)	78
TAX1BP1 (Q86VP1; 8887)	50.820 (31)	42
TOMM7 (Q9P0U1; 54543)	50.820 (31)	36
E2F1 (Q01094; 1869)	49.180 (30)	55
MUL1 (Q969V5; 79594)	49.180 (30)	62
AMBRA1 (Q9C0C7; 55626)	24.590 (15)	55
SRC (P12931; 6714)	18.033 (11)	37
TFEB (P19484 #; 7942)	9.836 (6)	58
HIF1 (Q16665; 3091)	8.197 (5)	51
PERK (Q9NZJ5; 9451)	6.557 (4)	63
OPTN (Q96CV9 #; 10133)	1.639 (1)	42

# ataxin-3 interactors for which docking using AlphaFold 3D protein structure predictions were not successful.

**Table 6:** Difference, in percentage, of the number of interfacing sites along the ataxin-3, when comparing expanded and WT ataxin-3 forms for the 46 mitophagy putative ataxin-3 interactors.

Gene Symbol (UniProtID; GeneID)	Percentage of Increase $
SAMM50 (Q9Y512; 25813)	34.211 (76; 50)
RAS (P62070; 22800)	31.148 (61; 42)
TOMM7 (Q9P0U1; 54543)	26.531 (49; 36)
BCL-xL (Q07817; 598)	26.389 (72; 53)
GP78 (Q9UKV5; 267)	25.000 (68; 51)
Parkin (O60260; 5071)	22.222 (63; 49)
FOXO3 (O43524; 2309)	21.154 (52; 41)
USP30 (Q70CQ3; 84749)	18.57 (70; 57)
MITF (O75030; 4286)	18.056 (72; 59)
C-JUN (P05412; 3725)	15.789 (76; 64)
MARCHF5 (Q9NX47; 54708)	15.714 (70; 59)
FIS1 (Q9Y3D6; 51024)	15.686 (51; 43)
TOMM40 (O96008; 10452)	15.254 (59; 50)
Beclin1 (Q14457; 8678)	15.152 (66; 56)
ARIH1 (Q9Y4X5; 25820)	11.765 (68; 60)
FUNDC1 (Q8IVP5; 139341)	8.475 (59; 54)
SIAH1 (Q8IUQ4; 6477)	8.333 (60; 55)
NDP52 (Q13137; 10241)	8.065 (62; 57)
P53 (P04637; 7157)	7.692 (65; 60)
TOMM70 (O94826; 9868)	5.660 (53; 50)
VCP (P55072; 7415)	5.634 (71; 67)
TAX1BP1 (Q86VP1; 8887)	5.556 (54; 51)
ATG9B (Q674R7; 285973)	5.556 (72; 68)
NBR1 (Q14596; 4077)	5.263 (57; 54)
PGAM5 (Q96HS1; 192111)	3.333 (60; 58)
MON1B (Q7L1V2; 22879)	1.754 (57; 56)
CK2 (P68400; 1457)	1.667 (60; 59)
SP1 (P08047; 6667)	0.000 (62; 62)
JNK (P45983; 5599)	0.000 (50; 50)
CITED2 (Q99967; 10370)	−1.538 (65; 66)
PHB2 (Q99623; 11331)	−1.786 (56; 57)
TOMM20L (Q6UXN7; 387990)	−2.128 (47; 48)
RABGEF1 (Q9UJ41; 27342)	−4.167 (48; 50)
NLRX1 (Q86UT6; 79671)	−9.091 (33; 36)
UBC (P62979; 6233)	−11.628 (43; 48)
BNIP3 (Q12983; 664)	−13.208 (53; 60)
RAB7B (Q96AH8; 338382)	−13.636 (44; 50)
MTX2 (O75431; 10651)	−14.894 (47; 54)
CCZ1 (P86790; 221960)	−17.778 (45; 53)
PINK1 (Q9BXM7; 65018)	−25.490 (51; 64)
BCL2L13 (Q9BXK5; 23786)	−25.806 (62; 78)
MFN2 (O95140; 9927)	−27.083 (48; 61)
NIX (O60238; 665)	−32.558 (43; 57)
TFE3 (P19532; 7030)	−36.842 (57; 78)
TBK1 (Q9UHD2; 29110)	−40.541 (37; 52)

$—increase, in percentage, of the number of interfacing residues when comparing expanded (first number within brackets) and WT (last number within brackets) ataxin-3 forms.

## Data Availability

The auto_p2docking results are available at Zenodo (https://doi.org/10.5281/zenodo.14761185).

## Supplementary Materials

The supporting information can be downloaded at: https://www.mdpi.com/article/10.3390/ijms26031325/s1.
