# Supplementary material for: Predicting Which Mitophagy Proteins Are Dysregulated in Spinocerebellar Ataxia Type 3 (SCA3) Using the Auto-p2docking Pipeline
Source: Int J Mol Sci. 2025 Feb 4;26(3):1325. doi: 10.3390/ijms26031325 (PMC11818632; doi:10.3390/ijms26031325)
Supplement: Supplementary file 1 [file ijms-26-01325-s001.zip › ijms-3372464-supplementary.pdf]

Predicting Which Mitophagy Proteins Are Dysregulated in Spinocerebellar Ataxia Type 3 (SCA3) Using the Auto-p2docking Pipeline

Jorge Vieira, Mariana Barros, Hugo López-Fernández, Daniel Glez-Peña, Alba Nogueira-Rodríguez, Cristina P. Vieira

Supplementary Material

Table S1. Gene symbol (UniProtKB number, GeneID) and description of the proteins assigned as involved in animal mitophagy in KEGG pathway database (https://www.genome.jp/kegg/pathway.html)

Gene (UniptotID; GeneID)	Description	Other genes of the same family
AMBRA1 (Q9C0C7; 55626)	autophagy and beclin 1 regulator 1	
ARIH1 (Q9Y4X5; 25820)	ariadne RBR E3 ubiquitin protein ligase 1	
ATF4 (P18848; 468)	activating transcription factor 4	
ATG9B (Q674R7; 285973)	autophagy related 9B	ATG9A (79065)
BCL-xL (Q07817; 598)	BCL-2-like 1	
BCL2L13 (Q9BXK5; 23786)	BCL-2-like protein 13	
Beclin1 (Q14457; 8678)	beclin 1	Beclin2 (441925)
BNIP3 (Q12983; 664)	BCL2 interacting protein 3	
CCZ1B (P86790; 221960)	CCZ1 homolog B, vacuolar protein trafficking and biogenesis associated complex	
c-Jun (P05412; 3725)	Jun proto-oncogene AP-1 transcription factor subunit	
CK2 (CSNK2A1) (P68400; 1457)	casein kinase 2 alpha 1	CCZ1 (51622)
CITED2 (Q99967; 10370)	Cbp/p300 interacting transactivator with Glu/Asp rich carboxy-terminal domain 2	
E2F1 (Q01094; 1869)	E2F transcription factor 1	
EIF-2A (P05198; 1965)	eukaryotic translation initiation factor 2 subunit alpha	CSNK2A2 (1459); CSNK2A3 (283106); CSNK2B (1460)
FIS1 (Q9Y3D6; 51024)	fission mitochondrial 1	
FKBP8 (Q14318; 23770)	FKBP prolyl isomerase 8	
FOXO3 (O43524; 2309)	forkhead box O3	
FUNDC1 (Q8IVP5; 139341)	FUN14 domain containing 1	
GP78 (Q9UKV5; 267)	glycoprotein 78	
HIF1 (Q16665; 3091)	hypoxia inducible factor 1 subunit alpha	
HUWE1 (Q7Z6Z7; 10075)	HECT, UBA and WWE domain containing E3 ubiquitin protein ligase 1	
JNK (MAPK8) (P45983; 5599)	c-Jun N-terminal kinase	MAPK9 (5601); MAPK10 (5602)
LC3 (GABARAP) (O95166¸11337)	microtubule-associated proteins 1A/1B light chain 3B	GABARAPL2 (11345); GABARAPL1 (23710); MAP1LC3BP1(392288); MAP1LC3C (440738); MAP1LC3B2 (643246); MAP1LC3B (81631); MAP1LC3A (84557)
MARCHF5 (Q9NX47; 54708)	membrane associated ring-CH-type finger 5	MON1A (84315)
MFN2 (O95140; 9927	mitofusin 2	MFN1 (mitofusin 1; 55669)
MITF (O75030; 4286)	melanocyte inducing transcription factor	
MON1B (Q7L1V2; 22879)	MON1 homolog B, secretory trafficking associated	

MTX2 (O75431; 10651)	metaxin 2	MTX1 (4580); MTX3 (345778)
MUL1 (Q969V5; 79594)	mitochondrial E3 ubiquitin protein ligase 1	
NBR1 (Q14596; 4077)	NBR1 autophagy cargo receptor	
NFKB (Q04206; 5970)	RELA proto-oncogene, NF-kB subunit	
NDP52 (Q13137; 10241)	calcium binding and coiled-coil domain 2	
NIX /BNIP3L (O60238; 665)	BCL2 interacting protein 3 like	
NLRX1 (Q86UT6; 79671)	NOD-like receptor X1	
OPA1 (O60313; 4976)	OPA1 mitochondrial dynamin like GTPase	
OPTN (Q96CV9; 10133)	optineurin	
P53 (P04637; 7157)	cytosolic tumor protein p53	
p62 /SQSTM1 (Q13501; 8878)	sequestosome 1	
Parkin (O60260; 5071)	Parkin	
PERK (Q9NZJ5; 9451)	translation initiation factor 2 alpha kinase 3	
PGAM5 (Q96HS1; 192111)	PGAM family member 5 serine/threonine protein phosphatase	
PHB2 (Q99623; 11331)	prohibitin 2	
PINK1 (Q9BXM7; 65018)	PTEN induced kinase 1	
RAB5A (P20339; 5868)	Ras-related protein Rab-5 member RAS oncogene family	RAB5B (5869); RAB5C (5878)
RAB7B (Q96AH8; 338382)	Ras-related protein Rab-7 member RAS oncogene family	RAB7A (7879)
RABGEF1 (Q9UJ41; 27342)	RAB guanine nucleotide exchange factor 1	
RAS (RRAS2) (P62070; 22800)	RAS related 2	HRAS (3265); KRAS (3845); NRAS (4893); RRAS (6237); MRAS (22808)
SAMM50 (Q9Y512; 25813)	SAMM50 sorting and assembly machinery component	
SIAH1 (Q8IUQ4; 6477)	siah E3 ubiquitin protein ligase 1	
SMURF1 (Q9HCE7; 57154)	SMAD specific E3 ubiquitin protein ligase 1	SMURF2 (64750)
SP1 (P08047; 6667)	SP1 transcription factor	
SRC (P12931; 6714)	SRC proto-oncogene, non-receptor tyrosine kinase	
TAX1BP1 (Q86VP1; 8887)	Tax1 binding protein 1	
TBC1D15 (Q8TC07; 64786)	TBC1 domain family member 15	TBC1D17 (79735)
TBK1 (Q9UHD2; 29110)	TANK binding kinase 1	
TFE3 (P19532; 7030)	transcription factor binding to IGHM enhancer 3	
TFEB (P19484; 7942)	transcription factor EB	
TOMM20L (Q6UXN7; 387990)	translocase of outer mitochondrial membrane 20 like	TOMM20 (9804)
TOMM40 (O96008; 10452)	translocase of outer mitochondrial membrane 40	TOMM40L (84134)
TOM7 (Q9P0U1; 54543)	translocase of outer mitochondrial membrane 7	
TOMM70 (O94826; 9868)	translocase of outer mitochondrial membrane 70	
TRAF2 (Q12933; 7186)	TNF receptor associated factor 2	
UBC (RPS27A) (P62979; 6233)	ribosomal protein S27a	UBA52 (7311); UBB (7314); UBC (7316)
ULK1 (O75385; 8408)	unc-51 like autophagy activating kinase 1	
USP15 (Q9Y4E8; 9958)	ubiquitin specific peptidase 15	
USP30 (Q70CQ3; 84749)	ubiquitin specific peptidase 30	
USP8 (P40818; 9101)	ubiquitin specific peptidase 8	
VCP (P55072; 7415)	valosin containing protein	

Table S2. Description of the modules used and analyses performed in the 12 protocols used in this work.

Protocol	Modules used	Analyses performed
1	*evoppi_querier*, *geneid2uniprotkb*, *human_prot_atlas*, *copy*, *intersect*	Obtains from EvoPPI the GeneIDs for all the genes encoding ataxin-3 interactors and intersect them with the provided list (68 GeneIDs involved in mitophagy). Converts GeneID in reference UniprotKB.<br>Retains those entries for which there is evidence of expression in the brain regions relevant for SCA3 (Brain_cerebral_cortex; Brain_basal_ganglia; Brain_thalamus; Brain_midbrain; Brain_cerebellum; Brain_pons; Brain_medulla_oblongata) using the Human Protein Atlas).
2	*Intersect*, *copy*, *geneid2uniprotkb*, *human_prot_atlas*	The list of GeneIDs obtained in protocol 1 are compared with the list of 68 GeneIDs involved in mitophagy. Converts these in reference UniprotKB numbers and keep only the proteins encoded by genes that are expressed in the brain regions relevant for SCA3 disease
3 and 5	*getalphafoldpdb*, *cport_like*, *consensus*, *haddock*, *pisa_ccp4_extract*, *pisa_server_extract*, *copy*	For the ligands obtained using protocols 1(3) or 2(5), 3D structures are retrieved from AlphaFold, using the UniprotKB lists obtained in protocol 1. Active and passive sites are estimated using scriber, scannet, ispred4 and sppider, and a consensus obtained. Protein-protein docking analyses are performed using HADDOCK and PISAePDB. The ataxin-3 3D structure prediction and active and passive sites obtained in Sousa e Silva et al. (2023) are used for the protein-protein docking analyses
4, 6, 8, 10, 12	*pisa_xml_extract*, *tabulate*, *get_pattern*, *highlight_regions*, *copy*	Tables are prepared based on the PISAePDB results obtained in protocols 3, 5, 7, 9, 11, respectively. Moreover, protein regions matching the pattern '111[01][01]1' are reported if they occur in more than 50% of the sequences. Such regions are highlighted in all protein complexes under analysis
7 and 9	*getditasserpdb*, *cport_like*, *consensus*, *haddock*, *pisa_ccp4_extract*, *pisa_server_extract*, *intersect*, copy	For the ligands present (7) not present (9) in EvoPPI that did not show an interaction with at least 50% of the interacting regions amino acid positions defined in Sousa e silva et al. 2023 (Run1), or for which HADDOCK could not find a docking solution (Run2), the analyses performed in protocol 3 were

		repeated but this time using 3D structures from D-I-TASSER database.
11	*haddock*, *pisa_ccp4_extract*, *pisa_server_extract*, *intersect*, copy	The expanded ataxin-3 structure and active and passive sites lists obtained in Sousa e Silva *et al*. (2023), are used for the protein-protein docking analyses. Only the AlphaFold or D-I-TASSER 3D structures that showed an interaction with at least 50% of the interacting regions amino acid positions defined in Sousa e Silva *et al*. (2023) were used. The lists of active and passive sites determined when running the corresponding projects were used.

Table S3. Running times according to the computer used (C1- Intel® Xeon® CPU ES-2695 v4 @2.10 GHz processor (72 CPUs) and 256 GB of RAM memory, C2- AMD® EPYC® 7401 24-Core @1.2 GHz processor (96 CPUs) and 1024 GB of RAM), and C3-AMD® EPYC® 7642 48-Core @2.3 GHz processor (192 CPUs), and 1024 GB of RAM) and size of the protein analysed using protocol 3, 5, 7, 9 and 11.

Protocol	Computer	Protein	Run time (minutes)	Size (amino acids)
3	C1	O43524	841	673
		O95166	187	117
		P05198	386	315
		P62070	290	204
		P68400	398	391
		Q85VP5	709	425
		Q96HS1	330	289
		Q99623	1100	299
3	C2	O60260	308	465
		O60313	785	960
		P20339	159	215
		Q6UXN7	183	152
		Q9HCE7	550	757
		Q9NX47	304	278
		Q9UKV5	452	646
		Q9Y3D6	160	152
		Q9Y512	418	469
3	C3	O94826	284	608
		O95140	506	757
		P04637	277	393
		P08047	371	785
		P40818	604	1118
		P55072	409	806
		P62979	154	156
		Q07817	155	233
		Q13501	292	440
		Q14457	523	450
5	C2	O75385	710	1050
		O75431	225	263
		P12931	374	536
		P86790	359	482
		Q12983	313	194
		Q14596	656	966
		Q7L1V2	382	547
		Q86UT6	609	975
		Q9UJ41	511	491
		O96008	357	361
		Q13137	295	446

		Q70CQ3	830	517
		Q86VP1	338	789
		Q8IUQ4	245	282
		Q969V5	347	352
		Q9C0C7	889	1298
		Q9P0U1	133	55
		Q9PXM7	489	37
		Q9UHD2	622	729
		Q9Y4E8	686	981
		Q9Y4X5	427	557
5	C3	O75030	316	526
		P18848	273	351
		P19532	655	575
		P45983	212	427
		Q01094	248	437
		Q16665	389	826
		Q8TC07	349	691
		Q96AH8	172	199
		Q99967	244	270
		Q9NZJ5	634	1116
7	C1	O43524	372	673
		O60313	1192	960
		O95166	429	117
		P05198	57	315
		P20339	382	215
		P40818	935	1118
		P62979	187	156
		Q07817	301	233
		Q13501	542	440
		Q68XN7	330	129
		Q99623	586	299
		Q9HCE7	466	757
		Q9Y3D6	158	152
		P05412	340	331
		Q04206	53	551
		Q12933	855	501
		Q14318	231	412
9	C1	O75030	336	526
		O75431	198	263
		P12931	346	536
		P45983	328	427
		Q01094	409	437
		Q12983	241	194
		Q16665	497	826

		Q70CQ3	338	517
		Q86VP1	588	789
		Q8IUQ4	220	282
		Q969V5	368	352
		Q9C0C7	1018	1298
		Q9NZJ5	638	1116
		Q9P0U1	119	55
		Q9UJ41	306	491
		Q9Y4X5	396	557
		O60238	161	219
		P19484	415	476
		Q674R7	455	924
		Q96CB9	530	384
		Q9BXK5	376	485
11*	C1	O96008	62	361
		P19532	1098	575
		P86790	376	482
		Q13137	508	446
		Q14596	1376	966
		Q7L1V2	476	547
		Q86UT6	702	975
		Q96AH8	257	199
		Q99967	457	270
		Q9BXM7	587	581
		Q9UHD2	784	729
11*	C2	O60260	262	465
		O94826	337	608
		O95140	718	757
		P04637	241	393
		P08047	504	785
		P55072	427	806
		P62070	183	204
		P68400	237	391
		Q8IVP5	232	155
		Q96HS1	431	289
		Q9NX47	484	278
		Q9UKB5	339	411
		Q9Y512	362	469
		Q14457	467	450
		O43524	445	673
		P05412	322	331
		P62979	120	156
		Q07817	202	233
		Q6UXN7	129	152

		Q99623	255	299
		Q9Y3D6	105	152
11*	C3	O60238	109	219
		Q674R7	321	924
		Q9BXK5	269	485
		O75030	227	526
		O75431	132	263
		P45983	201	427
		Q12983	160	194
		Q70CQ3	216	517
		Q86BP1	393	386
		Q8IUQ4	139	282
		Q9P0U1	78	55
		Q9VJ41	194	689
		Q9Y4X5	245	557

* not considering the pisa-ccp4-extract (about 7 minutes per PPI).
